# The Facial Skin Blood Flow Change of Stroke Patients with Facial Paralysis after Peripheral Magnetic Stimulation: A Pilot Study

**DOI:** 10.3390/brainsci12101271

**Published:** 2022-09-21

**Authors:** Yongli Zhang, Shugeng Chen, Yinglu Ruan, Jiaying Lin, Chengdong Li, Chong Li, Shuo Xu, Zhijie Yan, Xiangyun Liu, Peng Miao, Jie Jia

**Affiliations:** 1 School of Rehabilitation Medicine, Fujian University of Traditional Chinese Medicine, Fuzhou 350122, China; 2 Department of Rehabilitation Medicine, Huashan Hospital, Fudan University, Shanghai 200040, China; 3 School of Kinesiology, Shanghai University of Sport, Shanghai 200438, China; 4 Department of Rehabilitation Medicine, Shanghai Jing’an District Central Hospital, Shanghai 200040, China; 5 School of Biomedical Engineering, Shanghai Jiaotong University, Shanghai 200030, China; 6 National Clinical Research Center for Aging and Medicine, Huashan Hospital, Fudan University, Shanghai 200040, China; 7 National Center for Neurological Disorders, Shanghai 200040, China; 8 National Regional Medical Center, Fuzhou 350200, China

**Keywords:** facial paralysis, peripheral magnetic stimulation, blood flows, laser speckle, stroke

## Abstract

Background: Facial paralysis (FP) is a common symptom after stroke, which influences the quality of life and prognosis of patients. Recently, peripheral magnetic stimulation (PMS) shows potential effects on peripheral and central nervous system damage. However, the effect of PMS on FP after stroke is still unclear. Methods: In this study, we applied PMS on the facial nerve of nine stroke patients with FP. At the same time, laser speckle contrast imaging (LSCI) was used to explore the facial skin blood flow (SkBF) in 19 healthy subjects and nine stroke patients with FP before and after the PMS intervention. The whole face was divided into 14 regions to compare the SkBF in different sub-areas. Results: In baseline SkBF, we found that there were no significant differences in the SkBF between the left and right faces in the healthy subjects. However, there was a significant difference in the SkBF between the affected and unaffected faces in Region 7 (Chin area, *p* = 0.046). In the following five minutes after the PMS intervention (Pre_0–5 min), the SkBF increased in Region 5 (*p* = 0.014) and Region 7 (*p* = 0.046) and there was an increasing trend in Region 3 (*p* = 0.088) and Region 6 (*p* = 0.069). In the five to ten minutes after the intervention (Post_6–10 min), the SkBF increased in Region 5 (*p* = 0.009), Region 6 (*p* = 0.021) and Region 7 (*p* = 0.023) and there was an increasing trend in Region 3 (*p* = 0.080) and left and right whole face (*p* = 0.051). Conclusions: These pilot results indicate that PMS intervention could increase facial skin blood flow in stroke patients with FP. A further randomized controlled trial can be performed to explore its possible clinical efficacy.

## 1. Introduction

Central facial paralysis (CFP) is the common sequela for people who suffer from stroke, which affects about 45% of stroke patients according to a previous study [1]. CFP patients often present facial muscle dysfunction in the lower part of the face, which refers to the regions of the face below the eye clefts. It not only affects the facial appearance of patients but also causes some relevant deficits such as dysphagia and dysarthria [2,3]. Furthermore, patients with facial paralysis are more likely to have negative emotions like anxiety and depression than those who do not have facial paralysis [4]. Obviously, it may affect the training motivation of the CFP patients, which has a negative impact on clinical treatments. Thus, paying attention to facial paralysis after a stroke is important, as it influences the quality of life and prognosis of stroke patients [5].

In clinical practice, the common modalities for treating facial paralysis include orofacial exercises [6], mirror therapy [7], acupuncture [8], electrical stimulation [9], and cupping [10]. Training in which the patient actively activates his facial muscles is called active rehabilitation, such as orofacial training. Training in which the patient accepts stimulation passively is called passive rehabilitation, such as acupuncture. Unlike skeletal muscles of limbs, facial muscles contain smaller motor units [11,12]. It seems difficult for patients to control facial movements precisely. Thus, applying active rehabilitation training alone might not achieve satisfactory efficacy. A passive and effective method as a complementary therapy is needed in treating facial paralysis better [13,14].

In recent years, neurostimulation techniques have been widely used for stroke patients in motor and sensory function rehabilitation [15]. For example, electrical stimulation is a common-used technique for facial paralysis in stroke patients [2]. Lee et al. [16] tried to determine the positive effect of neuromuscular electrical stimulation (NMES) on the masseter muscle in acute stroke patients. The results showed that it had a therapeutic effect on oral dysfunction in post-stroke patients. OH et al. [17] applied NMES to stroke patients on their orbicularis oris muscle and they found the lip strength and lip closure function of patients were significantly improved. Choi [2] et al. verified the effect of NMES on improving facial muscle strength and oral function in stroke patients with facial paralysis. However, the potential for painful feelings and allergic reactions to the electrodes during treatment limits the further application of electrical stimulation [18,19]. Peripheral magnetic stimulation (PMS) has been proposed as an alternative to electrical stimulation [20] as it generates a magnetic field and causes eddy currents inside the body, which is similar to the operating principle of electrical stimulation. It could directly penetrate deeper structures and tissues [21] with a less painful sensation of the skin [22] and no risk of inducing allergic reactions.

As a noninvasive treatment method, PMS induces muscle contractions and sensory afferents and it is considered to be an effective treatment in individuals with stroke or other nerve disorders [23,24,25]. The underlying mechanism of PMS in stroke rehabilitation has been explored for years. There were studies [26,27] speculating that by using PMS on hemiplegia limbs, the activation of frontoparietal loops promoted the improvement of patients’ motor function. Besides, it was found that the increased corticomotor excitability of the lesioned hemisphere might be one of the key points [28]. Although PMS has been shown to improve the motor function of the upper and lower limbs in post-stroke patients [14,29], it has not yet been used in treating the facial dysfunction of stroke patients.

Most post-stroke patients suffer from motor dysfunction and it is mostly manifested as increased muscle tension, decreased muscle strength, and decreased blood flow [30,31]. Blood flow is one of the most important objective indicators for motor recovery. Several studies have demonstrated that motor function is related to tissue perfusion or blood flow in stroke patients [32,33]. As a general evaluation tool, laser speckle contrast imaging (LSCI) technology is recognized as a useful tool to determine skin blood flow (SkBF). It is a method of using the speckle pattern created after a laser strikes the moving red blood cells [34]. A unique edge of LSCI is that it provides real-time imaging to monitor near-continuous flow and it is a non-invasive method [35]. According to the previous study, LSCI is recommended for use as an important quantitative tool in clinical studies [36].

As a pilot study, this study aims to compare the facial skin blood flow of healthy subjects and stroke patients with facial paralysis. As for stroke patients, the whole face was divided into 14 regions to explore the SkBF characteristics of different facial areas. The pre-intervention SkBF and post-intervention SkBF were compared to reflect the peripheral effects of a single peripheral magnetic stimulation. Moreover, a customized questionnaire was offered to patients and it was a window to reflect the patients’ real feelings and acceptance of PMS. We hypothesized that the blood flow would increase on the affected face of stroke patients after the PMS intervention.

## 2. Materials and Methods

### 2.1. Patients Recruitment

This study was performed from March 2022 to April 2022. Nine stroke patients (male, *n* = 5) with central facial paralysis and 19 healthy subjects (male, *n* = 5) without organic diseases were enrolled in the Department of Rehabilitation Medicine, Shanghai Jing’an District Central Hospital. The inclusion criteria of stroke patients were: (1) diagnosed with first unilateral hemispheric stroke confirmed by computed tomography (CT), and/or magnetic resonance imaging (MRI); (2) stroke onset after two weeks; (3) with unilateral central facial paralysis; (4) have not been administered drugs like corticosteroids for facial paralysis. The exclusion criteria were: (1) obvious wounds on faces; (2) diseases that affect blood flow except for hypertension (e.g., metabolic diseases or limb edema); (3) without enough cognitive ability to complete the study. Table 1 lists the patients’ demographic and baseline clinical characteristics.

The study received approval from the Ethics Committee of Shanghai Jing’an District Central Hospital (KY2022-06), and each participant or his/her authorized representatives provided written informed consent for study participation. This study was registered in the Chinese Clinical Trial Registry (ChiCTR2200057805).

### 2.2. Measurement of Facial Skin Blood Flow

In this study, prototype imaging equipment was used to measure the skin’s blood flow. It includes a CCD camera (acA1300-60 gmNIR, Basler AG, Ahrensburg, Germany) and a height-adjustable vertical stand. By starting measurements, a diverging near-infrared laser beam with a wavelength of 830 nm was used to illuminate the imaging area. The reflected light was then collected by the CCD camera with a frame rate of 30 fps and resolution of 640 × 480 (binning 2 × 2). The distance between the laser head and the skin surface was kept at 25 cm [37] and the imaging area was about 30 cm × 30 cm in size.

**Figure 1:**
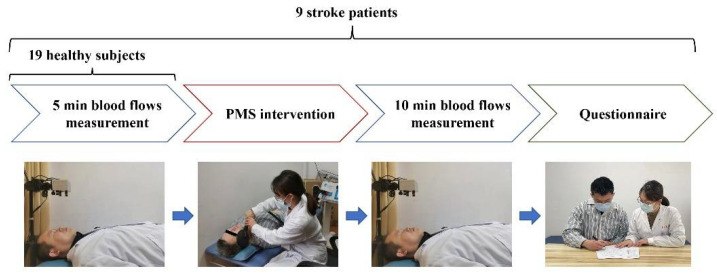
Experimental setup and intervention protocol.

All the participants, including the healthy subjects and stroke patients, received the facial skin blood flow measurement. The healthy subjects received five minutes of facial blood flow measurement and did not take PMS intervention. Stroke patients sequentially underwent a 5-min pre-intervention blood flow measurement and a 10-min post-intervention blood flow measurement. Figure 1 presents the experimental setup.

Before starting the SkBF measurement, both healthy subjects and stroke patients were asked to rest for 20 min in an air-conditioned room which is maintained at around 22 °C and 40–60% humidity. In addition, the measurements were performed under normal fluorescent lighting conditions. During measurements, the participants were asked to lie down on the examination bed and close their eyes. They were also asked to keep their faces neutral and not to make facial movements. SkBF measurement was performed as soon as the single PMS intervention finished.

### 2.3. PMS Intervention

In this study, only stroke patients received PMS intervention. By using a Mag-Pro R30 magnetic device (Tonica Elektronik A/S, Farum, Denmark) and a coil (MMC-140, Tonica Elektronik A/S), PMS intervention was administered. With the special parabola shape, the coil generates focused stimulation. The inner diameter of the coil is 25 mm and the outer diameter is 120 mm. Each stimulation was set as 20 Hz for 1.5 s and the intervals of every two stimulations were set at 3 s. The intensity level of magnetic stimulation was set at 15% of the maximal stimulator output for the device. In total, the entire intervention took about 20 min (1215 s) and produced 270 cycles of stimulation (8100 pulses). During the intervention, the patients maintained the supine position, and they were asked to keep relaxing. The stimulating coil was placed over the affected face of the FP patients and kept perpendicular to the skin to stimulate the outlet of the facial nerve and its branches of the lower part of the face (zygomatic branch, buccal branch, and mandibular branch).

### 2.4. The Facial Regions of Interest (ROIs)

**Figure 2:**
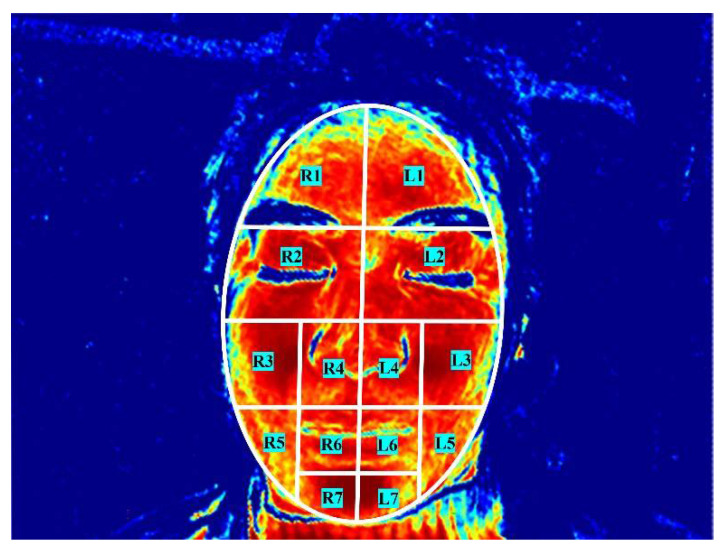
The facial regions of interest. L, left; R, right.

In this study, the whole face was divided into 14 regions of interest (ROIs) based on previous research [38,39]. The 14 regions can represent the characteristics of different parts of the face in FP patients. There are seven ROIs on each side and are the frontal region (L1/R1), ocular region (L2/R2), infraorbital region and zygomatic region (L3/R3), nasal region (L4/R4), buccal region (L5/R5), lip region (L6/R6) and the chin region (L7/R7). All the ROIs are distributed in pairs on the left and right sides of the face (Figure 2).

### 2.5. Safety Section

In order to reflect the patients’ treatment experience of PMS, a questionnaire including five subcomponents (tolerance, comfort, preference, pain, and numbness) was designed and applied. A seven-point Likert scale was used, and “1” means “strongly disagree” and “7” means “strongly agree” in the questionnaire. After the post-intervention blood flow measurements, the questionnaire and an open-ended question was answered by the patients. The question is “Use one word to explain your main feelings during intervention” (Question 1). Furthermore, on the next day after the PMS intervention, patients were requested to answer another question “Use one word to explain your intervention-related feelings today” (Question 2).

### 2.6. Statistics

We used P1 to stand for 0–5 min before the PMS intervention, P2 for 0–5 min after the PMS intervention, P3 for 6–10 min after the PMS intervention, and P4 for 0–10 min after the PMS intervention. Averaged SkBF was calculated for P1, P2, P3, and P4. A *t*-test was conducted to compare the differences in SkBF between the healthy subjects and stroke patients. It is also applied to analyze the skin blood flow in the left and right sides of the faces of healthy subjects and the skin blood flow in the affected and unaffected faces of stroke patients. A paired *t*-test was adopted for the comparison before (P1) and after the PMS intervention (P2, P3, and P4) in stroke patients. Analyses were conducted using SPSS version 23.0 (IBM Inc., Chicago, IL, USA). Data of skin blood flow and scores of five subcomponents of the questionnaire are presented as the mean ± standard deviation. A *p*-value of <0.05 (two-sided) was considered to indicate a significant result.

## 3. Results

### 3.1. Left and Right Facial Skin Blood Flow of Healthy and Stroke with FP

Table 2 shows the facial skin blood flow between healthy subjects and stroke patients with facial paralysis. There were no significant differences in the SkBF between the left and right faces of the seven regions and both half faces in the healthy subjects. There were also no significant differences in SkBF between the affected and unaffected face except for a significant difference in Region 7 (*p* = 0.046) between the affected (219.32) and unaffected (231.56) face in the stroke patients with FP. The facial skin blood flow in Region 7 of the affected face was lower than that on the unaffected face.

Compared to the SkBF of the left (197.44) or right (198.14) face of the healthy subjects (*p* = 0.695), the facial skin blood flow of the stroke patients (*p* = 0.289) on the affected face (193.60) was lower while that of the unaffected face (201.30) was higher. However, no significant differences were found between the two groups.

### 3.2. Left and Right Facial Skin Blood Flow before and after PMS in Stroke with FP

Table 3 shows the facial skin blood flow before and after a single PMS intervention in stroke patients with FP. Before the intervention, the SkBF on the affected face (both on the half face and seven regions) of all patients was lower than that on the healthy side. After the intervention, the SkBF on the affected face (both on the half-face and seven regions) in stroke patients all increased and it even exceeded that of the unaffected side in some areas (half face, Region 2, Region 3, Region 4, Region 5, Region 6, Region 7). There was no statistical difference between the affected and unaffected sides after the PMS intervention, but it shows a trend. For the affected and unaffected half face, the facial skin blood flow changed from 193.60 (P1) to 203.62 (P2) and 205.58 (P3) on the affected face, and from 201.30 (P1) to 200.51 (P2) and 205.13 (P3) on the unaffected face.

### 3.3. The Change of Facial Skin Blood Flow before and after PMS in Stroke with FP

**Figure 3:**
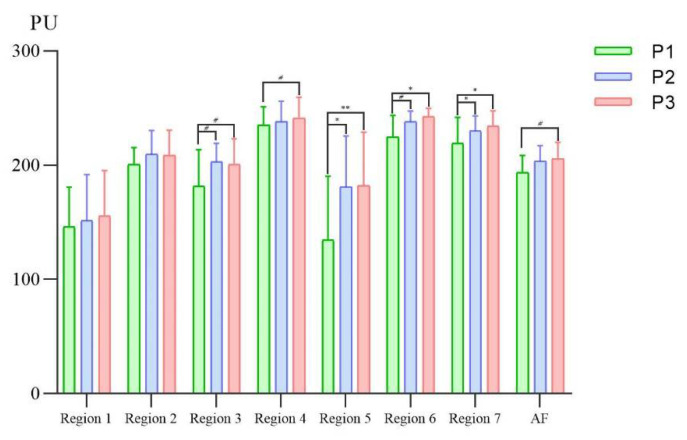
Bar plots of facial skin blood flow of the affected face before and after peripheral magnetic stimulation in stroke patients with facial paralysis. P1, 0–5 min before the PMS intervention; P2, 0–5 min after the PMS intervention; P3, 6–10 min after the PMS intervention; AF, the affected face; PU, perfusion index. #, 0.05 < *p* < 0.1; *, 0.01 < *p* < 0.05; **, *p* < 0.01.

**Figure 4:**
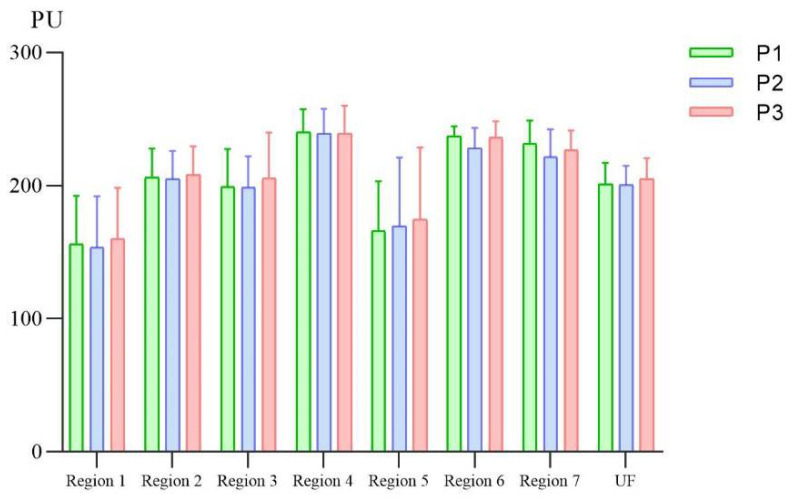
Bar plots of facial skin blood flow of the unaffected face before and after peripheral magnetic stimulation in stroke patients with facial paralysis. P1, 0–5 min before the PMS intervention; P2, 0–5 min after the PMS intervention; P3, 6–10 min after the PMS intervention; UF, the unaffected face; PU, perfusion index.

Table 4 shows the statistical differences between pre-PMS and post-PMS intervention in stroke patients with facial paralysis. On the affected face in stroke patients, the facial skin blood flow increased significantly in Region 5 (*p* = 0.014) and Region 7 (*p* = 0.046) and there was an increasing trend in Region 3 (*p* = 0.088) and Region 6 (*p* = 0.069) before (P1) and after (P2) the PMS intervention. Furthermore, the SkBF on the affected face of patients increased in Region 5 (*p* = 0.009), Region 6 (*p* = 0.021) and Region 7 (*p* = 0.023) significantly, and there was an increasing trend in Region 3 (*p* = 0.080) and left and right face (*p* = 0.051) before (P1) and after (P3) the PMS intervention (Figure 3). However, there was no significant difference in the SkBF on the unaffected face of stroke patients between pre-PMS and post-PMS intervention (Figure 4).

### 3.4. Safety Section

Of the nine stroke patients, six felt muscle twitch, one reflexed vibration sensation, one felt uncomfortable with the noise of the equipment, and one reported numbness in Question 1. In Question 2, four patients reported an increase in muscle strength subjectively, two with a feeling of skin tightening, one with a feeling of skin lifting, and two with no special feeling on the next day after PMS intervention. Table 5 shows the results of the questionnaire. The scores of the five subcomponents are tolerance (6.33 ± 0.71), comfort (5.11 ± 1.36), preference (6.56 ± 0.73), pain (1.44 ± 1.33), and numbness (4.11 ± 2.20), respectively. Patients reported high tolerance and almost no pain during and after the PMS intervention.

## 4. Discussion

In this study, we compared the face characteristics of SkBF in 19 healthy subjects and nine stroke patients with FP. The difference in SkBF between healthy subjects and FP patients was analyzed. Furthermore, a single session of PMS was applied to the nine stroke patients with facial paralysis. Through the change of SkBF before and after the PMS intervention, we initially explored the peripheral effects of PMS. The SkBF of 0 to 5 min and 6 to 10 min after the intervention were compared to find the continuous effect of PMS. Moreover, a questionnaire was offered to the patients to test irsafety and to explore their feelings and acceptance of PMS.

### 4.1. Facial Blood Flow on Both Sides of the Participants

Our results showed no statistical difference between the left side and right side in the facial skin blood flow of healthy subjects. The result was the same as several previous studies [40,41]. As for the stroke patients, some studies suggested that the blood flow of stroke patients on the affected limbs was lower than on the unaffected side. Tiftik et al. [33] showed that the radial and ulnar arteries of the affected side were significantly smaller in volume flow and end-diastolic velocity. Billinger et al. [42] found that the blood flow was also reduced in the femoral artery of the affected body. A major reason for this is likely a lack of physical activity and the disuse of the limbs [32]. Our study firstly explored the facial skin blood flow of both faces in stroke patients and showed similar conclusions, of which the SkBF of the affected side was lower than the unaffected side. However, the differences in SkBF in most of the regions were not statistically significant (R1, R2, R3, R4, R5, R6). This might be related to the anatomy of the face. Unlike the limbs, the face cannot complete facial movement with unilateral muscles, but with the coordinated movement of both sides. It might reduce differences in the blood flow on both sides.

Compared to healthy subjects, the incidence of stroke affects the motor performance of stroke patients. Some studies speculated that it might be related to the hemodynamic changes after the stroke onset [32]. Murphy et al. [43] found that a lower magnitude of muscle perfusion through the femoral artery was observed in the stroke group than in the neurologically intact participants. Zhang et al. [36] demonstrated that superficial perfusion of hands which was measured by laser speckle contrast imaging was lower than healthy volunteers. In our study, compared to healthy volunteers, we also found the affected side face of stroke patients had a lower skin blood flow.

### 4.2. The Effects of PMS on Facial Skin Blood Flow

Normal muscle function requires a certain amount of SkBF. Decreased perfusion after stroke has been suggested as one of the possible reasons for the impact on muscle performance [36]. Some studies have demonstrated that with the advancement of treatment, motor recovery is accompanied by an increase in blood perfusion [32]. It may suggest the potential of blood perfusion as one of the indicators of exercise improvement. As a pilot study, the outcome measurement was the key to exploring the effectiveness because the common evaluation indicators were hard to reflect the change of a single intervention. A real-time evaluation indicator is thus needed. Unlike the study mentioned above which applied long-term intervention, we chose the facial skin blood flow as the index to monitor the change in muscle status.

In our study, patients presented a higher facial skin blood flow after the PMS intervention. The SkBF in Region 5 and Region 7 both significantly increased in P2 and P3 compared to P1. It is likely that the continuous stimulation of PMS leads to passive facial muscle movement in stroke patients which contributed to the increase in blood flow. Furthermore, the major symptom of central facial paralysis patients is mouth drooping. That is one of the reasons why Region 5 and Region 7 (related regions) have obvious changes compared to other regions [44]. The SkBF in Region 3 maintained an increasing trend in both P2 and P3. Interestingly, as time went on, the SkBF in Region 6 changed from an increasing trend in P2 to a significant increase in P3 compared to P1, which suggested an accumulative effect of the PMS intervention [45]. Besides, there was also an increasing trend in the affected face which covered Region 1 to Region 7. This suggested the activation effect on SkBF from the PMS intervention [28]. Previous studies [46,47,48] considered that both peripheral magnetic and electrical stimulations can have a modulating and activating effect, which would lead to improvements in sensorimotor function. Additionally, the possible mechanisms that may underlay regional differences in facial SkBF are the autonomic control, and local control of vasomotion in facial skin vessels [49]. That means when facing different physiological or psychological stimulation, human faces present unique responses in facial regions [50].

### 4.3. The Acceptance of the Facial PMS Intervention

According to the questionnaires, patients had a high tolerance and comfort during the PMS intervention. They felt little pain caused by the magnetic stimulation. However, some patients reported a feeling of mild numbness. It might be due to the stimulation affecting the surrounding nerve (trigeminal nerve), which is mainly responsible for facial sensation. Overall, patients had a higher preference for PMS compared to other treatments, which suggested the high acceptance of PMS. In addition, through the open-ended questions, we found that the immediate feeling during treatment and the subsequent feeling the next day after intervention were not the same. During PMS stimulation, most patients felt the twitching of the muscles. On the next day, most patients felt an increase in muscle strength on the affected side. It might be caused by the legacy effects of PMS stimulation on the receptors.

There are some limitations to be noted in this study. First of all, the sample size was small, which consisted of only nine stroke patients and 19 healthy subjects. With a larger sample, it might help to eliminate outliers and present robust data better. Furthermore, although we performed a single PMS intervention, it could not reflect the efficacy of peripheral magnetic stimulation in improving facial function. Indeed, a direct effect would take several weeks to occur [45 s].

## 5. Conclusions

This study demonstrated that the facial skin blood flow increased on the affected face of stroke patients with facial paralysis after a single session of PMS intervention. The findings of this study extended the possible application of PMS and preliminarily verified the feasibility of applying PMS to stroke patients with facial paralysis.

## Figures and Tables

**Table 1:** The demographic and baseline clinical characteristics of the stroke patients with facial paralysis.

Patients	Sex	Age (y)	Type of Injury	Affected Face	Time Post-Stroke (Month)	House-Brackmann
S1	Male	46–50	hemorrhage	Right	11	IV
S2	Male	66–70	ischemia	Right	4	III
S3	Male	51–55	ischemia	Right	4	II
S4	Male	46–50	hemorrhage	Left	52	IV
S5	Female	66–70	ischemia	Left	6	III
S6	Female	71–75	hemorrhage	Right	2	II
S7	Female	66–70	ischemia	Right	1	II
S8	Female	51–55	hemorrhage	Right	105	II
S9	Male	71–75	ischemia	Left	5	IV

**Table 2:** The facial skin blood flow between healthy subjects and stroke patients with facial paralysis.

	Healthy Subjects (*n* = 19)			Stroke Patients (*n* = 9) Pre-Intervention		
Region	Left	Right	*p* Value	Affected	Unaffected	*p* Value
1	151.00	151.36	0.947	145.89	156.43	0.139
2	203.18	201.86	0.659	200.52	206.09	0.378
3	209.23	215.95	0.142	181.84	199.50	0.286
4	233.10	230.75	0.616	235.45	240.20	0.192
5	191.90	191.16	0.931	134.45	166.05	0.181
6	237.20	236.17	0.639	225.00	237.11	0.130
7	232.16	238.53	0.099	219.32	231.56	0.046
half face	197.44	198.14	0.695	193.60	201.30	0.289

**Table 3:** The facial skin blood flow between the affected and unaffected faces in stroke patients with facial paralysis.

	Pre_0–5 min (P1)			Post_0–5 min (P2)			Post_6–10 min (P3)			Post_0–10 min (P4)		
Region	Affected	Unaffected	*p* Value	Affected	Unaffected	*p* Value	Affected	Unaffected	*p* Value	Affected	Unaffected	*p* Value
1	145.89	156.43	0.139	151.87	153.96	0.665	155.63	160.35	0.268	154.01	156.81	0.501
2	200.52	206.10	0.378	209.56	205.15	0.079	208.71	208.44	0.959	209.21	206.54	0.435
3	181.84	199.50	0.286	203.11	198.85	0.581	200.96	205.54	0.783	202.48	201.86	0.958
4	235.45	240.20	0.192	238.36	239.37	0.496	241.08	239.47	0.284	239.71	239.41	0.820
5	134.45	166.05	0.181	181.21	169.77	0.457	182.27	174.71	0.633	182.72	172.68	0.509
6	225.00	237.11	0.130	238.16	228.50	0.054	242.69	236.06	0.124	240.61	232.49	0.064
7	219.32	231.55	0.046	230.06	221.67	0.116	234.33	226.93	0.170	232.53	224.50	0.107
half face	193.60	201.30	0.289	203.62	200.51	0.306	205.58	205.13	0.919	204.81	202.66	0.510

**Table 4:** The statistical differences of the facial skin blood flow between pre-PMS and post-PMS intervention in stroke patients with facial paralysis. P1, 0–5 min before the PMS intervention; P2, 0–5 min after the PMS intervention; P3, 6–10 min after the PMS intervention; P4, 0–10 min after the PMS intervention.

	Affected Side			Unaffected Side		
Region\Time	P1-P2	P1-P3	P1-P4	P1-P2	P1-P3	P1-P4
1	0.483	0.289	0.362	0.579	0.357	0.929
2	0.150	0.195	0.152	0.823	0.599	0.921
3	0.088	0.080	0.072	0.937	0.538	0.792
4	0.273	0.067	0.115	0.773	0.833	0.800
5	0.014	0.009	0.010	0.778	0.533	0.617
6	0.069	0.021	0.035	0.173	0.832	0.388
7	0.046	0.023	0.023	0.107	0.403	0.197
half face	0.126	0.051	0.077	0.838	0.395	0.744

**Table 5:** Results of the questionnaire.

Subcomponent (Description)	Scores (Mean ± SD)
**Tolerance** (I accepted the treatment and stick to finish it until the end easily for me)	6.33 ± 0.71
**Comfort** (The treatment process is comfortable)	5.11 ± 1.36
**Preference** (If the treatment effects are the same, I would prefer peripheral magnetic stimulation to other facial treatments)	6.56 ± 0.73
**Painful** (During the treatment, I felt pain in the treated area)	1.44 ± 1.33
**Numbness** (During the treatment, I felt numbness)	4.11 ± 2.20

## Data Availability

Not applicable.
